# P-1713. Impact of an Evidence-Based Order Panel on Antimicrobial Prescribing in Ambulatory Patients with Complicated and Uncomplicated Cystitis

**DOI:** 10.1093/ofid/ofae631.1879

**Published:** 2025-01-29

**Authors:** Matt Neumann, Kelsey L Jensen, Ryan W W Stevens, Dan Ilges, Abinash Virk, Paschalis Vergidis, Kristin Mara

**Affiliations:** Mayo Clinic Health System, Austin, Minnesota; Mayo Clinic Health System - Southeast Minnesota, Osage, Iowa; Mayo Clinic, Rochester, MN; Mayo Clinic Arizona, Phoenix, Arizona; Mayo Clinic, Rochester, MN; Mayo Clinic, Rochester, MN; Mayo Clinic, Rochester, MN

## Abstract

**Background:**

Optimizing antimicrobial prescribing for urinary tract infections (UTI) represents an area of opportunity for ambulatory antimicrobial stewardship programs. A pre-populated, ambulatory antibiotic order panel for UTI was implemented in the Mayo Clinic Enterprise on 5/16/2022. The order panel provides antimicrobial regimens which align with both institutional and national treatment guidelines based on patient comorbidities, complicating factors, and beta-lactam allergy status. We aimed to assess the impact of panel use on antibiotic prescribing for complicated and uncomplicated cystitis in the ambulatory setting.

**Figure:**
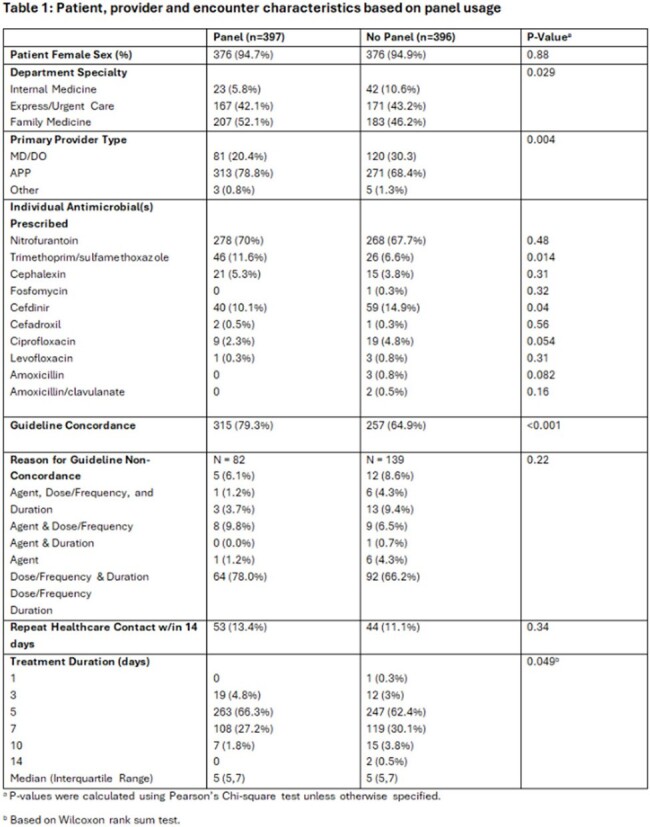

**Methods:**

This retrospective review included ambulatory encounters from the Mayo Clinic Enterprise in 2022 and 2023. Eligible encounters were randomly selected and included those with a primary ICD-10 code of cystitis performed by primary care or urgent care clinicians. Encounters were excluded if patients had symptoms indicative of pyelonephritis, underwent urinary instrumentation within 30 days, or had a urinary pathogen isolated within 90 days. The primary outcome was concordance with institutional guidelines. Secondary outcomes included repeat healthcare contact and therapy duration. Outcomes were analyzed with Pearson's chi-squared and Wilcoxon rank sum tests (α level = 0.05).

**Results:**

793 encounters (397 panel vs. 396 non-panel) were included. Prescribing was guideline concordant in 79.3% and 64.9% (p< 0.001) of panel and non-panel encounters, respectively. Inappropriate duration was the most common reason for non-concordance in the panel use cohort. There was no difference between groups for repeat healthcare contact incidence. Antimicrobials prescribed differed between panel and no-panel groups (Table 1), with higher use of sulfamethoxazole/trimethoprim and lower use of cefdinir in the panel cohort. Numerically shorter durations were seen in the panel group, with more 3- and 5-day regimens than the no-panel group.

**Conclusion:**

Use of a pre-populated antimicrobial order panel for outpatient cystitis encounters resulted in greater adherence to institutional guideline recommendations, particularly in terms of antimicrobial selection, without adversely impacting repeat healthcare contact.

**Disclosures:**

**Paschalis Vergidis, MD, MSc**, AbbVie: Advisor/Consultant|Ansun Biopharma: Grant/Research Support|Cidara Therapeutics: Grant/Research Support|F2G: Grant/Research Support|Scynexis: Advisor/Consultant|Scynexis: Grant/Research Support

